# Aligning Minds in Spasticity Care—A Two-Phase Delphi-Dialogue Study of Patients and Professionals in Spain

**DOI:** 10.3390/toxins18010056

**Published:** 2026-01-22

**Authors:** Helena Bascuñana-Ambrós, Jacobo Formigo-Couceiro, José Maria Climent-Barberá, Lluis Guirao-Cano, Michelle Catta-Preta, Alex Trejo-Omeñaca, Josep Maria Monguet-Fierro

**Affiliations:** 1 Physical Medicine and Rehabilitation Department, Sant Pau University Hospital, 08041 Barcelona, Spain; 2 Spanish Society of Physical Medicine and Rehabilitation (SERMEF), 28016 Madrid, Spain; 3 Physical Medicine and Rehabilitation Department, A Coruña University Hospital, 15006 A Coruña, Spain; 4 Rehabilitation Research Group, Biomedical Research Institute of A Coruña (INIBIC), 15006 A Coruña, Spain; 5 Physical Medicine and Rehabilitation Department, Mutua de Terrassa University Hospital, 08221 Terrassa, Spain; 6 Innex Labs, 08800 Vilanova i la Geltrú, Spainjm.monguet@upc.edu (J.M.M.-F.); 7 Escola Politècnica Superior d’Enginyeria de Vilanova i la Geltrú (EPSEVG), Universitat Politècnica de Catalunya, 08041 Barcelona, Spain

**Keywords:** spasticity, botulinum toxin, patient-centered care, rehabilitation, Real-Time Delphi, consensus

## Abstract

Background: Spasticity, which occurs with certain neurological conditions, substantially affects quality of life, function, and social participation. Despite widespread botulinum toxin use, variability persists in patient information, access to specialized rehabilitation, and follow-up models. Methods: This two-phase Delphi-Dialogue Patients–Professionals study (DDPP), promoted by SERMEF, integrated perspectives from 77 patients and 141 rehabilitation professionals. Phase 1 used parallel surveys to assess satisfaction, perceived effectiveness of botulinum toxin, communication preferences, and rehabilitation follow-up. Phase 2 applied Real-Time Delphi with 38 experts to generate consensus recommendations to improve spasticity management. Results: Patients and professionals agreed on botulinum toxin benefits, the importance of ongoing rehabilitation, and the value of hybrid (in-person/remote) follow-up. Key gaps concerned access to Physical Medicine and Rehabilitation services, clarity and timing of information, and shared goal setting. Experts translated these misalignments into 10 prioritized recommendations, with highest feasibility for actions standardizing access pathways, optimizing botulinum toxin use, reinforcing structured education, and consolidating hybrid rehabilitation models. Conclusions: The DDPP approach offers a reproducible framework to align stakeholder perspectives by converting quantified divergence into consensus-based innovation priorities. Implementing the recommendations—particularly those strengthening communication, education, and hybrid pathways regarding botulinum toxin treatment—may support more accessible, personalized, patient-centered spasticity care.

## 1. Introduction

Spasticity is a motor disorder that arises due to central nervous system lesions and is manifested as a velocity-dependent increase in resistance to passive stretch, associated with positive signs such as clonus, spasms, and exaggerated reflexes and negative features such as weakness and loss of selective motor control. Lance’s classical definition has been widely used in clinical practice; however, more recent approaches expand its scope. Dressler et al. proposed redefining spasticity within the broader concept of involuntary muscle overactivity associated with upper motor neuron lesions, particularly in the context of botulinum toxin therapy [1]. Similarly, Mukherjee and Chakravarty emphasized that spasticity results from complex pathophysiological mechanisms that clinicians must consider in daily practice [2].

Spasticity is common across neurological conditions. After stroke, systematic reviews estimate a prevalence of approximately 25% overall, with higher rates in specific cohorts [3]. It is also highly prevalent in spinal cord injury and cerebral palsy, where spastic forms comprise nearly 80% of cases [4]. Spasticity contributes to pain, contractures, mobility limitations, and reduced quality of life, creating a substantial socioeconomic burden. Recent reviews have also highlighted the importance of improving quantitative assessment methods to better characterize and monitor spasticity, which may guide treatment selection and research in novel interventions [5].

Management requires a multimodal approach. Botulinum toxin type A (BoNT-A) remains the cornerstone for focal spasticity, with demonstrated efficacy across etiologies and muscle groups [4]. Complementary interventional strategies are increasingly used in complex or refractory cases. Phenol neurolysis has long been applied in focal patterns, with contemporary surveys and cohort studies describing its practice patterns and feasibility under ultrasound and electrostimulation guidance [6,7]. Diagnostic nerve blocks (DNBs) with local anesthetics are gaining recognition as valuable tools to distinguish neural overactivity from fixed contracture and to optimize BoNT-A treatment goals [8].

In recent years, minimally invasive ablative procedures have been explored. Cryoneurotomy and cryoneurolysis represent emerging options capable of inducing reversible axonotmesis with documented reductions in tone and functional gains in post-stroke and long-standing spasticity [9,10]. Similarly, ultrasound-guided radiofrequency thermal neuroablation has been reported as a promising technique for refractory adductor and rectus femoris spasticity [11], and its application to the musculocutaneous nerve has also been reported as a safe and effective option for patients with severe spasticity presenting an elbow flexor pattern [12]. For pain management, particularly in hemiplegic shoulder pain, intra-articular BoNT-A injections have shown positive results in reducing pain and improving mobility [13].

Recently non-invasive adjunctive therapies have gained growing relevance in the management of spasticity, among which Extracorporeal Shock Wave Therapy (ESWT) has emerged as a promising option. A recent systematic review and meta-analysis including 42 studies and 1973 patients concluded that ESWT significantly reduces Modified Ashworth Scale scores in individuals with upper motor neuron lesions, although the methodological quality of available trials remains limited and heterogeneity is high [14]. Likewise, a more recent systematic review focused on adults with stroke and cerebral palsy—encompassing 16 studies—found that ESWT effectively reduced spasticity in both upper and lower limbs, with effects maintained up to 12 weeks after treatment [15]. However, controversy persists regarding the magnitude of effect, the most effective wave type (radial vs. focal), optimal stimulation parameters, and duration of benefits, underscoring the need for larger, high-quality randomized controlled trials to establish standardized protocols and long-term efficacy.

While emerging modalities such as ESWT expand the therapeutic toolbox, their real-world impact ultimately depends on whether treatment goals, expected benefits, and follow-up plans are explicitly co-defined with patients and revisited over time.

Beyond technical advances, patient participation in the rehabilitation process has become a central paradigm. A systematic review by Rose et al. demonstrated that shared decision-making during rehabilitation goal-setting enhances patient satisfaction and engagement [16]. More recently, longitudinal evidence confirmed that active patient involvement in rehabilitation processes is associated with better functional outcomes and higher goal attainment [17]. Such findings support integrating shared decision-making into spasticity management, ensuring that treatment strategies are not only clinically effective but also aligned with patients’ priorities.

Nevertheless, implementing shared decision-making consistently in routine spasticity care is challenged by variability in access to specialized services, differences in referral pathways, and heterogeneous follow-up models across settings [18,19,20]. These organizational constraints can widen patient–professional misalignment by limiting structured education and opportunities to recalibrate expectations (including perceived waning of BoNT-A effects), and by fragmenting continuity between injection visits and rehabilitation delivery [21,22,23]. In parallel, the expansion of telemedicine and hybrid pathways introduces additional sources of divergence related to digital literacy, trust, and patient preferences, which require clear protocols and clinician oversight to preserve safety and personalization [24,25,26,27,28].

Finally, in the European context, consensus initiatives have emphasized the need for patient-centered criteria in selecting advanced therapies such as intrathecal baclofen or BoNT-A, underscoring that therapeutic strategies should consider both clinical suitability and patient preferences [4].

Taken together, these considerations point to a clear knowledge gap: despite therapeutic progress, structured evidence remains limited on where—and why—patients and clinicians diverge in expectations, priorities, communication needs, and perceptions of follow-up across the spasticity care pathway, and which service-level changes are both meaningful and feasible to implement. Addressing this gap is essential to move from technically effective interventions to consistently patient-centered, equitable models of care.

## 2. Objectives

### 2.1. General Aim

The primary aim of this study is to establish shared priorities for improving long-term care in Chronic Brain Injury (CBI) rehabilitation by integrating the perspectives of patients, rehabilitation professionals, and clinical experts through a structured consensus-based approach.

### 2.2. Specific Objectives

1. To systematically capture and compare patient and professional experiences in CBI rehabilitation, identifying areas of convergence and divergence regarding current models of care, communication practices, and perceived quality of services.
2. To assess attitudes toward digital and hybrid rehabilitation models, including tele-rehabilitation and remote communication tools, from both patient and professional perspectives.
3. To evaluate the feasibility of targeted improvement strategies for CBI rehabilitation services through an expert-driven Real-Time Delphi process, prioritizing interventions that are realistic and actionable within current healthcare settings.

## 3. Results

This study identified significant differences in the perceptions and experiences of spasticity rehabilitation between patients and professionals, while also achieving consensus on recommendations to improve services. Across the two phases of the process, perceptions of care quality were systematically analyzed, and proposals were prioritized according to their feasibility of implementation. These findings culminated in a final proposal organized into specific innovation domains aimed at strengthening patient-centered spasticity care.

### 3.1. Comparison of Experiences Between Patients and Professionals

The differences in perception of the different items evaluated are shown in Table 1. Those differences are calculated in the following manner: A synthetic indicator “d” is proposed, combining the standardized difference between perceptions (SDI) with the high-agreement gap (ΔA), thus capturing both the magnitude of the discrepancy and its consensual relevance.

Formally:

d = SDI × ΔA

where

- SDI = |μ_pat_ − μ_pro_|/√[(σ^2^_pat_ + σ^2^_pro_)/2];
- ΔA = |(A_pat_ − A_pro_)/Min (A_pat_; A_pro_)|;
- μ_pat_, μ_pro_: for patients and professionals;
- σ_pat_, σ_pro_: standard deviations for patients and professionals;
- A_pat_, A_pro_: Sum of answers 5 and 6 for patients and professionals.

Interpretation:

- d = 0: perfect alignment—similar perceptions and comparable high agreement.
- d increases as the difference in perception grows and high-agreement convergence decreases, divergence rises.

### 3.2. Second Phase: Validation and Prioritization of Recommendations

**Figure 1:**
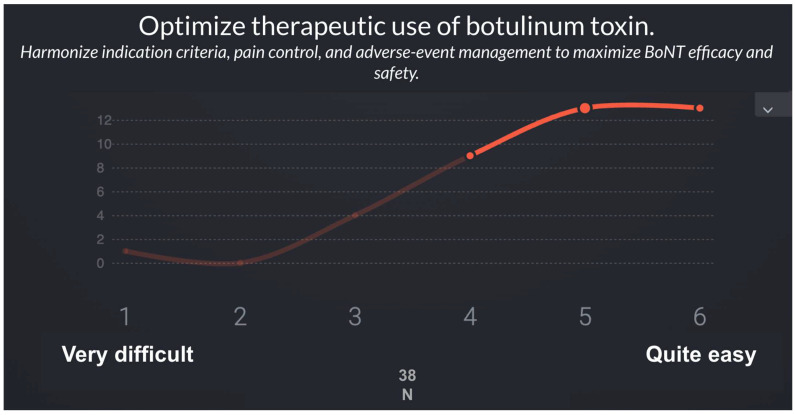
Sample screen of the Smart Delphi tool used to facilitate consensus in the Real-Time Delphi synchronous focus group.

In the second phase, a panel of 38 specialists in Physical Medicine and Rehabilitation (PM&R) with expertise in spasticity evaluated the feasibility of implementing the recommendations derived from Phase 1 using a Real-Time Delphi process (Figure 1). Table 2 and Table 3 summarize the list of recommendations and the results of the Real-Time Delphi process.

**Figure 2:**
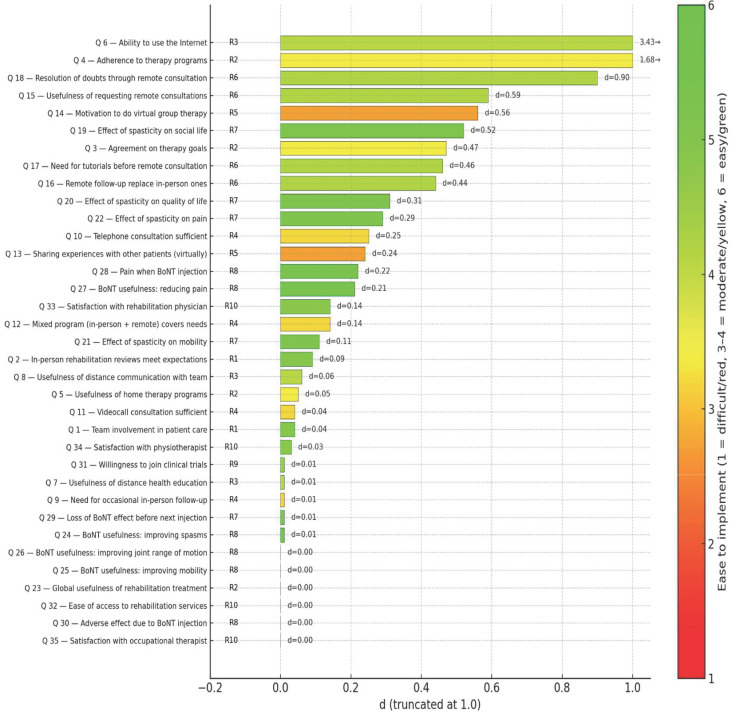
Divergence (“d”) of survey items and ease of implementation of corresponding recommendations. Each bar represents the standardized divergence index (d) for one survey item, ordered from lowest to highest. Bar colors reflect the ease of implementation (Likert 1–6) of the linked recommendations: red = difficult, yellow = moderate, and green = easy. Codes on the left (R1–R10) indicate the corresponding recommendations, while the numerical labels to the right of each bar show the exact d values.

In the second phase, the panel of 38 spasticity experts assessed the feasibility of implementing the recommendations proposed using a Real-Time Delphi application. Table 3 presents the 10 recommendations proposed with their feasibility scoring on a 1–6 Likert scale. Recommendations are ordered from least to most difficult to implement considering the statistical variables (μ, σ, Med, IQR). The Figure 2 represents the combination of recommendations and survey items. 

### 3.3. Final Proposal: Innovation Domains (ID) in Spasticity Rehabilitation

Building on the previous findings, the research team consolidated the 10 recommendations into four strategic innovation domains that reflect both the Delphi consensus and the thematic structure of the subsequent discussion.

ID1. Access, coordination, and communication in rehabilitation care.

Strengthening interdisciplinary coordination and ensuring timely, traceable communication between patients and professionals emerged as the first priority. Standardizing referral pathways and promoting structured dialog—supported by written and audiovisual materials—can improve transparency, continuity, and patient confidence across all stages of the rehabilitation process.

ID2. Education and sustained adherence.

Structured educational strategies for patients and caregivers are essential to promote understanding, engagement, and long-term adherence. Implementing clear therapeutic plans, self-management guides, and pre-visit tutorials for remote sessions can reinforce motivation and bridge the gap between professional prescription and patient follow-through.

ID3. Digital transition and hybrid rehabilitation.

Integrating in-person and remote care through hybrid models offers flexibility without compromising therapeutic quality. Prioritizing digital health literacy, developing safe tele-rehabilitation environments, and formalizing remote follow-up protocols will facilitate the gradual adoption of digital practices aligned with patient capabilities and preferences.

ID4. Personalization and innovation in therapeutic pathways.

Optimizing botulinum toxin (BoNT) treatment through individualized schedules, predictive models, and continuous feedback mechanisms represents a key opportunity for innovation. These advances, together with participatory approaches that value patient experience, can transform technological progress into genuine cultural change toward a more inclusive, patient-centered rehabilitation model.

These four domains form the conceptual basis for the following discussion, which examines how communication, digital transition, therapeutic optimization, and organizational culture jointly define the future of patient-centered spasticity care.

## 4. Discussion

### 4.1. Contribution Beyond Existing Evidence

This study provides a structured and participatory assessment of chronic brain injury (CBI) rehabilitation by integrating patient experiences, professional perspectives, and expert consensus. Unlike prior studies that focus primarily on clinical effectiveness or technological feasibility, the main contribution of this work lies in translating experiential data into prioritized and feasible directions for service improvement.

The clinical effectiveness of key rehabilitation interventions, including botulinum toxin treatment, as well as the potential role of tele-rehabilitation in chronic neurological conditions, has been widely documented [18,19,20,21,22,23,24]. Similarly, the importance of patient education, communication, and self-management in chronic care models is well established [16,25]. The present study does not aim to reassess this evidence. Instead, it advances the field by identifying how patients and professionals differ in their perceptions and priorities, and by demonstrating how these differences can be systematically addressed through a structured consensus-based methodology.

### 4.2. Divergences and Convergences Between Patients and Professionals

The results reveal consistent divergences between patients and professionals, particularly regarding perceived team involvement, continuity of care, and acceptance of telemedicine as a substitute for face-to-face visits. Professionals generally reported higher levels of engagement and readiness for digital solutions, whereas patients expressed greater caution, especially in relation to replacing in-person encounters.

At the same time, strong convergence was observed in areas related to communication, education, and support for hybrid models of care. These convergent priorities provided a stable foundation for the expert consensus phase, ensuring that feasibility assessments were grounded in shared concerns rather than isolated viewpoints. As previously reported, barriers related to access, coordination, and service organization remain a persistent challenge in spasticity care [20,21,22].

### 4.3. Access and Communication Gaps

Nearly 40% of patients reported difficulties accessing Physical Medicine and Rehabilitation (PM&R) services or understanding their therapeutic plan, while most professionals considered their involvement sufficient. This mismatch mirrors the structural barriers reported internationally regarding the limited availability of trained providers, referral variability, and uneven continuity between hospital and community phases [20,21]. Similar findings have been described in consensus statements of the American Academy of Physical Medicine and Rehabilitation (AAPM&R) and in large, real-world surveys of spasticity care [21,22]. Addressing these inequalities requires coordinated system-level actions: standardized referral pathways, tele-expertise shared between hospitals, and structured education for both professionals and patients [18,23,24].

Communication and patient education emerge as central determinants of satisfaction and adherence. Educational interventions and structured dialog have shown measurable improvement in engagement and self-management capacity [16,25]. Yet, despite the availability of evidence-based BoNT guidelines [21,26,27], patients often report insufficient information about treatment intervals, expected outcomes, and side effects. Reinforcing bidirectional, traceable communication—through written plans, audiovisual material, and shared-decision tools—should therefore be a standard component of multidisciplinary spasticity management.

### 4.4. Integrated Interpretation of Tele-Rehabilitation, Education, and Communication

Rather than treating tele-rehabilitation, patient education, and communication as independent domains, the findings support their interpretation as interdependent components of hybrid rehabilitation models. While previous work has shown that tele-rehabilitation can be clinically comparable to face-to-face interventions in selected contexts [28,29,30], our results highlight that acceptance depends strongly on how these tools are embedded within care pathways.

Experts rated structured educational resources, reference videos, and formalized hybrid consultations as more feasible than fully bidirectional telemedicine or informal communication channels. This aligns with prior evidence supporting structured self-management and educational strategies [16,25] while emphasizing that incremental integration within existing workflows is more acceptable than disruptive replacement of in-person care.

Beyond general calls for cultural or systemic change, the study yields several concrete implications. First, hybrid rehabilitation models should be formally recognized within service planning rather than treated as temporary or exceptional solutions. Second, structured educational materials—including audiovisual resources—should be systematically incorporated into rehabilitation pathways to support continuity and self-management. Third, transitional moments such as hospital discharge require clearer role definition and follow-up mechanisms to reduce patient-perceived fragmentation.

Communication tools should be institutionally supported and aligned with healthcare system standards, avoiding reliance on informal platforms that may compromise governance or equity. These implications extend existing guideline-based recommendations for patient-centered rehabilitation and spasticity management [21,26,27].

### 4.5. Therapeutic Personalization and Optimization of BoNT

Both patients and professionals recognized BoNT as effective and well tolerated, in agreement with systematic reviews and pragmatic clinical studies [18,19,20,21,22,23,24]. Nonetheless, a substantial proportion of patients perceived a waning of therapeutic effect before the next scheduled injection, reflecting variability in pharmacodynamics and muscle response.

Evidence supports individualized injection intervals based on functional goals and severity of spasticity [26,27]. Moreover, combined programs integrating BoNT treatment with stretching, physical therapy, or home-based exercises have been shown to amplify and prolong therapeutic gains [29,31]. The use of ultrasound-guided injections and appropriate governance frameworks further enhances precision and safety in clinical practice [26].

### 4.6. Methodological Contribution and Replicability of the DDPP Approach

A key strength of this study is the Delphi-Dialogue Patients–Professionals (DDPP) methodology itself. Unlike traditional Delphi studies focused exclusively on expert opinion, the DDPP framework integrates experiential data from patients and professionals prior to consensus building. Its modular structure—parallel surveys, concordance analysis, and real-time Delphi—allows adaptation to other chronic conditions and rehabilitation contexts without reliance on disease-specific protocols.

Replication would primarily require stakeholder engagement, digital survey infrastructure, and expert facilitation, rather than substantial technological investment. In this sense, the methodology offers a scalable tool for participatory innovation in chronic care, complementing existing evidence rather than duplicating it.

## 5. Conclusions

This study demonstrates the value of the Delphi-Dialogue Patients–Professionals (DDPP) methodology as a structured and participatory approach for guiding innovation in chronic brain injury (CBI) rehabilitation. Beyond describing current practices, the study provides a systematic framework to integrate patient experiences, professional perspectives, and expert judgment into a coherent set of prioritized and feasible recommendations.

A key contribution of this work lies in its ability to make divergences visible and actionable. While patients and professionals broadly share goals related to improving long-term rehabilitation, they differ in their perceptions of team involvement, communication quality, and the role of digital and remote care. By explicitly identifying these gaps and translating them into consensus-based priorities, the DDPP approach moves beyond traditional needs assessments and supports evidence-informed service planning.

The study also highlights that hybrid rehabilitation models—combining face-to-face care with structured digital and educational support—represent a realistic and acceptable pathway for innovation. Importantly, the findings suggest that successful implementation depends less on technological sophistication than on organizational clarity, continuity of care, and patient-centered communication strategies.

Several methodological limitations should be acknowledged. The Delphi process, while effective in building consensus, reflects expert opinion rather than empirical implementation outcomes. In addition, the use of online tools and structured surveys may have influenced participation and response patterns, potentially underrepresenting individuals with lower digital literacy. The national context and the professional composition of the sample may further limit generalizability.

Despite these limitations, the DDPP methodology offers a transferable and scalable model for participatory innovation in rehabilitation and other chronic care settings. Future work should focus on piloting the prioritized recommendations in real-world clinical environments, evaluating their impact on patient outcomes and service efficiency, and extending the methodology to multicenter and international contexts.

## 6. Materials and Methods

### 6.1. Study Design and Context

This was a sequential, mixed-methods observational consensus study conducted in two complementary phases designed to explore and align patient and professional perspectives on spasticity care.

Phase 1 involved parallel cross-sectional surveys administered to two target groups: (1) adults living with spasticity, and (2) rehabilitation professionals (physicians, physiotherapists, and occupational therapists) with expertise in spasticity management. The aim was to systematically map their experiences, perceptions, and unmet needs throughout the rehabilitation process.

Phase 2 consisted of a Real-Time Delphi process conducted to prioritize the recommendations emerging from Phase 1 and to assess their feasibility for implementation in clinical practice. This participatory stage enabled consensus building between patients and professionals, focusing on actionable innovations in care delivery. The software used in this phase was SmartDelphi 2025 (smartdelphi.com). 

Fieldwork for Phase 1 was carried out between January and May 2023, while the Real-Time Delphi session took place in May 2024.

Recruitment of rehabilitation physicians was conducted nationwide through the Spanish Society of Physical Medicine and Rehabilitation (SERMEF), ensuring broad geographical representation across Spain. Patients were recruited through their treating rehabilitation physicians in hospitals throughout the national territory, including individuals receiving ongoing treatment for spasticity.

**Figure 3:**
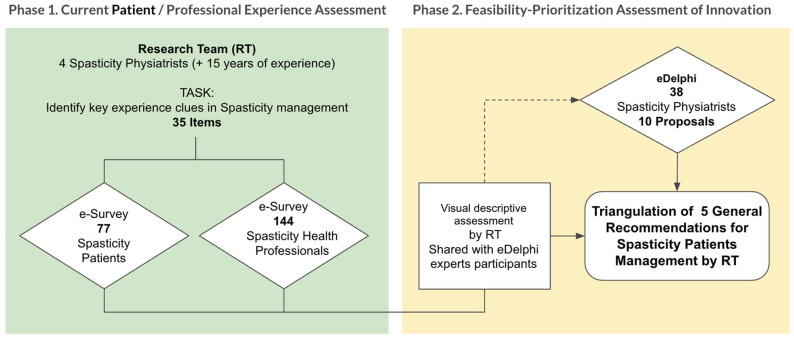
Flow of the two phases of the research. (RT: research team).

Figure 3 illustrates the overall study workflow—from survey distribution to the generation and prioritization of recommendations—as well as the analytic framework applied to integrate patient and professional perspectives.

### 6.2. Participants and Inclusion Criteria

#### 6.2.1. General Inclusion Criteria

Patients. Adults (≥18 years old) with a confirmed diagnosis of spasticity of any etiology—including stroke, traumatic brain injury, spinal cord injury, or cerebral palsy—were eligible for participation, provided they had prior exposure to rehabilitation care and consented voluntarily to take part in the study.

Professionals. Participants included rehabilitation physicians, physiotherapists, and occupational therapists with active clinical involvement in the assessment and management of spasticity within hospital or specialized outpatient settings. National recruitment was coordinated through the Spanish Society of Physical Medicine and Rehabilitation (SERMEF) to ensure wide geographical representation across Spain.

#### 6.2.2. Data Collected

Demographic and profile variables were recorded for both groups to contextualize their responses.

For patients, data included age, sex, underlying etiology of spasticity, functional impact, and rehabilitation trajectory (time since diagnosis, treatment follow-up, and exposure to botulinum toxin therapy) (Table 1).

For professionals, data included sex, years of clinical experience treating spasticity, type of healthcare institution, and involvement in academic or research activities (Table 2).

Table 4 summarizes the characteristics of the patients and Table 5 summarizes the characteristics of the professionals.

### 6.3. Questionnaires

Two parallel online questionnaires (patients/professionals) were specifically designed for this study, each structured into three sections (Table 1, Table 2 and Table 3):

1. Profile and characterization variables:
  - Ten items for patients and 7 items for professionals describing demographic and clinical background (Table 1 and Table 2).
2. Common items:
  - Eighteen questions addressing shared domains such as access to rehabilitation services, adequacy of clinical information, involvement of the multidisciplinary rehabilitation team, use of hybrid or tele-rehabilitation models, and preferred communication channels (Table 3).
3. Condition-specific items:
  - Seventeen questions focused on spasticity care, including botulinum toxin (BoNT) treatment itineraries (perceived utility, waning effects, adverse events) and the functional and social impact of spasticity.

All items were rated using a six-point Likert scale (1 = very little/poor; 6 = very much/excellent). The inclusion of a common core of 18 items enabled direct comparison between patients and professionals, whereas the condition-specific modules provided a deeper understanding of spasticity-related issues and treatment perceptions.

### 6.4. Procedure

The study followed a two-phase sequential workflow, combining quantitative and consensus-building methodologies to progressively refine priorities and generate actionable recommendations.

Phase 1: Surveys

Invitations were disseminated through professional networks of the Spanish Society of Physical Medicine and Rehabilitation (SERMEF) and national patient associations. Participants accessed the surveys through a secure online platform that ensured anonymous participation and data protection. Eligibility was verified through screening questions confirming (a) active clinical involvement in spasticity care for professionals and (b) a confirmed diagnosis of spasticity with prior exposure to rehabilitation for patients. Responses were automatically coded and aggregated for analysis, preventing any personal identification.

Phase 2: Real-Time Delphi

Building on the areas of convergence and divergence identified in Phase 1, the research team synthesized a preliminary list of candidate recommendations addressing key domains such as: pre- and post-BoNT education; adjustment of injection intervals and “rescue” access; bidirectional and traceable communication pathways and, hybrid and digitally supported follow-up models.

Professionals participated in a Real-Time Delphi session, where they rated each proposed recommendation for implementation feasibility using a six-point Likert scale (1 = very little/poor; 6 = very much/excellent). Immediate visual feedback and controlled mini-iterations within the same round were applied to enhance the stability and robustness of the consensus process.

The variables assessed in each phase are described below.

Phase 1 (Surveys)

- Primary outcome: alignment gap between patients and professionals across domains: access, information, BoNT treatment, communication and follow-up.
- Indicators: mean scores, proportion of agreement (≥5/6), and Synthetic Indicator (d) combining the standardized difference between perceptions and the agreement gap.

**Figure 4:**
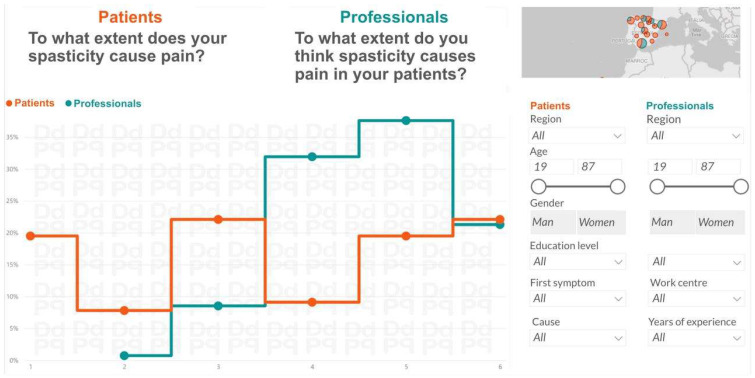
Sample screen of the visual descriptive analysis to compare the answers to the same survey questions from patients and from professionals. This visualization was shared with experts prior to the synchronous Real-Time Delphi to assess recommendations.

The research team prepared a visual presentation of the comparative analysis of the two surveys, patients and professionals (Figure 4), to prepare the work of experts in Phase 2.

Phase 2 (Real-Time Delphi)

- Indicators: mean (μ), standard deviation (σ), median (Med) and interquartile range (IQR).
- Operational output: prioritized list of recommendations by feasibility (μ, Med, IQR) with complementary notes for clinical implementation.

We applied descriptive statistics (means, standard deviations, proportions, confidence intervals where applicable) and comparative analyses (differences in means and agreement proportions between groups). Concordance was summarized using the Synthetic Indicator(d).

For the Real-Time Delphi phase, recommendations were ordered by feasibility parameters (μ, Med, IQR).

## Figures and Tables

**Table 1:** Differences in perception between patients and professionals.

		Patients			Professionals				
ID		μ	σ	A	Μ	σ	A	SDI	d
Q 6	Ability to use the Internet	4.4	1.82	58.5	3.3	0.94	10.6	0.76	3.43
Q 4	Adherence to therapy programs	4.4	1.42	57.2	3.6	0.93	16.3	0.67	1.68
Q 18	Resolution of doubts through remote consultation	2.9	1.48	11.7	3.7	1.13	29	0.61	0.90
Q 15	Usefulness of requesting remote consultations	3.4	1.66	26	4.3	1.33	51.8	0.60	0.59
Q 14	Motivation to do virtual group therapy	3.2	1.68	23.4	4.2	1.20	42.5	0.69	0.56
Q 19	Effect of spasticity on social life	4.5	1.22	55.9	5.4	0.73	88.6	0.89	0.52
Q 3	Agreement on therapy goals	4.1	1.64	44.2	5	0.88	74.5	0.69	0.47
Q 17	Need for tutorials before remote consultation	3.4	1.72	31.2	4.4	1.32	53.2	0.65	0.46
Q 16	Remote follow-up can replace in-person ones	2.6	1.59	15.6	3.5	1.40	26.9	0.60	0.44
Q 20	Effect of spasticity on quality of life	4.9	1.05	66.3	5.6	0.65	91.5	0.81	0.31
Q 22	Effect of spasticity on pain	3.7	1.81	41.6	4.7	0.92	58.9	0.70	0.29
Q 10	Telephone consultation sufficient	3.2	1.55	18.2	2.9	1.16	8.5	0.22	0.25
Q 13	Sharing experiences with other patients (virtually)	3.6	1.76	33.8	4.4	1.05	48.2	0.55	0.24
Q 28	Pain when BoNT injection	2.8	1.64	18.2	3.3	1.11	11.3	0.36	0.22
Q 27	BoNT usefulness: reducing pain	4.3	1.71	53.3	4.9	0.83	78.7	0.45	0.21
Q 12	Mixed program (in-person + remote) covers needs	4.1	1.65	44.2	4.7	1.08	58.8	0.43	0.14
Q 33	Satisfaction with rehabilitation physician	5.2	1.30	81.8	4.7	0.81	63.1	0.46	0.14
Q 21	Effect of spasticity on mobility	4.6	1.41	58.5	5	0.76	77.3	0.35	0.11
Q 2	In-person rehabilitation reviews meet expectations	4.8	1.36	67.6	4.5	0.80	51.1	0.27	0.09
Q 8	Usefulness of distance communication with team	4.2	1.65	45.5	4	1.12	32.6	0.14	0.06
Q 5	Usefulness of home therapy programs	4.2	1.54	46.8	4.6	1.07	53.9	0.30	0.05
Q 1	Team involvement in patient care	5	1.23	76.7	5.3	0.77	86.5	0.29	0.04
Q 11	Videocall consultation sufficient	3.5	1.65	27.3	3.4	1.22	17.8	0.07	0.04
Q 34	Satisfaction with physiotherapist	4.9	1.48	72.8	4.6	0.85	65.3	0.25	0.03
Q 24	BoNT usefulness: improving spasms	4.8	1.54	65	4.9	0.95	75.2	0.08	0.01
Q 29	Loss of BoNT effect before next injection	4.4	1.58	58.5	4.5	0.98	50.4	0.08	0.01
Q 9	Need for occasional in-person follow-up	4.4	1.48	57.2	4.6	0.95	61	0.16	0.01
Q 7	Usefulness of distance health education	3.8	1.61	39	4.1	1.14	40.4	0.22	0.01
Q 31	Willingness to join clinical trials	4.2	1.94	55.9	4.3	1.13	50.3	0.06	0.01
Q 35	Satisfaction with occupational therapist	4.3	1.85	58.5	4.4	1.13	55.1	0.07	0.00
Q 30	Adverse effect due to BoNT injection	1.8	1.43	76.6	2	0.80	77.3	0.17	0.00
Q 32	Ease of access to rehabilitation services	4	1.91	49.4	4.2	1.08	49.7	0.13	0.00
Q 23	Global usefulness of rehabilitation treatment	5.3	1.10	83.1	5.2	0.84	83.7	0.10	0.00
Q 25	BoNT usefulness: improving mobility	4.6	1.62	65	4.8	1.01	65.2	0.15	0.00
Q 26	BoNT usefulness: improving joint range of motion	4.8	1.44	67.6	4.8	0.91	70.2	0.00	0.00

**Table 2:** Recommendations to innovate patient-centered spasticity care.

No.	Recommendation	Linked Survey Items	Description
1	Strengthen interdisciplinary coordination in rehabilitation care	1–2	Formalize team workflows to ensure coherent, goal-oriented care during hospitalization and outpatient care.
2	Ensure adherence and continuity of rehabilitation programs	3–5, 23	Use shared goal-setting, structured home programs, and tailored education/monitoring to sustain adherence over time.
3	Promote digital health literacy and safe technology use in rehabilitation	6–8	Provide training and support for patients and staff to integrate tele-rehabilitation, remote education, and secure communication.
4	Implement hybrid follow-up models (in-person + telemedicine)	9–12	Implement flexible mixed care pathways that preserve therapeutic quality while optimizing time and resources.
5	Reinforce the social and motivational dimension of virtual therapy	13–14	Add structured peer-interaction and group dynamics in digital environments to enhance engagement and motivation.
6	Standardize remote follow-up with protocols and support tools	15–18	Define pre-visit tutorials, operational protocols, and patient-reported feedback loops to evaluate impact and satisfaction.
7	Develop predictive models of spasticity course	19–22, 29	Build analytics linking spasticity severity, function, and BoNT waning to anticipate needs and personalize follow-up.
8	Optimize therapeutic use of botulinum toxin	24–28, 30	Harmonize indication criteria, pain control, and adverse-event management to maximize BoNT efficacy and safety.
9	Foster patient participation in research and continuous improvement	31	Facilitate enrollment in clinical studies and co-design initiatives to accelerate evidence generation and validation.
10	Improve accessibility and global satisfaction with rehabilitation services	32–35	Address access barriers and monitor satisfaction across disciplines to strengthen quality and continuity of care.

Abbreviations: BoNT: botulinum toxin.

**Table 3:** Real-Time Delphi prioritization of recommendations to promote patient-centered spasticity care.

N	Question	μ	σ	Med	IQR
R8	Optimize therapeutic use of botulinum toxin	5.3	1.0	5	1
R7	Develop predictive models of spasticity course	5.1	0.9	5	1
R1	Strengthen interdisciplinary coordination in rehabilitation care	4.9	1.2	5	1
R10	Improve accessibility and global satisfaction with rehabilitation services	4.8	1.3	5	2
R9	Foster patient participation in research and continuous improvement	4.5	1.2	5	2
R6	Standardize remote follow-up with protocols and support tools	4.2	1.1	4	2
R3	Promote digital health literacy and safe technology use in rehab	4.1	1.5	4	3
R2	Ensure adherence and continuity of rehabilitation programs	3.4	1.3	4	2
R4	Implement hybrid follow-up models (in-person + telemedicine)	3.2	1.4	3	2
R5	Reinforce the social and motivational dimension of virtual therapy	2.7	1.3	2	3

N indicates the original order in the Real-Time Delphi survey.

**Table 4:** Characteristics of participating patients (*n* = 77).

Variable	Distribution
Gender	Male: 57.1% (*n* = 44); Female: 42.9% (*n* = 33)
Education	Secondary/Vocational: 45.5%; University: 31.2%; Primary: 19.5%; No formal education: 3.9%
Living situation	Independent at home (51.9%);·Family caregiver (36.4%); Professional caregiver (3.9%); Supervised housing (3.9%); Nursing home (3.9%)
Etiology of spasticity	Ischemic stroke: 27.3%; Hemorrhagic stroke: 15.6%; Cerebral palsy: 11.7%; Multiple sclerosis: 10.4%; Spinal cord injury: 10.4%; Traumatic brain injury: 7.8%; Other: 16.9%
Time from symptom onset to diagnosis	<1 year: 32.5%; 1–5 years: 33.8%; 6–10 years: 11.7%; >10 years: 22.1%
Time from diagnosis to botulinum toxin treatment	<1 month: 11.7%; 1–3 months: 18.2%; 4–6 months: 20.8%; >6 months: 49.4%
Treatments received	Botulinum toxin: 85.9%; Physiotherapy: 57.8%; Occupational therapy: 31.2%; Orthoses: 28.1%; Oral medication: 20.3%; Nerve blocks: 12.5%
Employment impact	Previously employed: 62.3%; Currently employed: 22.1%; Work disability recognized: 57.1% (of which 54.5% due to spasticity)
Comorbidities	Chronic disease in addition to spasticity: 46.8%
Housing adaptation	Adapted: 57.1%; Not adapted: 42.9%

**Table 5:** Characteristics of participating professionals (*n* = 141).

Variable	Distribution
Gender	Female: 77.3% (*n* = 109); Male: 22.7% (*n* = 32)
Professional category	PM&R physicians: 86.5%; Physiotherapists: 8.5%; Occupational therapists: 5%
Workplace	Referral hospital: 61%; Area/Secondary; hospital (~400 beds): 25.5%; Basic general; hospital (~200 beds): 5%; District hospital (~100 beds): 5.7%; Other: 2.8%
Experience in spasticity	<5 years: 17.7%; 6–10 years: 24.1%; 11–15 years: 18.4%; 15–20 years: 22%; >20 years: 17.7%
Experience in interventional management of spasticity	<5 years: 26.2%; 6–10 years: 28.4%; 11–15 years: 17.7%; 15–20 years: 16.3%; >20 years: 11.3%
Treatments used	Physiotherapy 95%; Botulinum toxin 90.1%; Orthoses 87.9%; Occupational therapy 67.4%; Oral medication 61%; Nerve blocks 37.6%; Intrathecal baclofen pump 24.1%; Shock wave therapy 19.9%; Radiofrequency 7.8%
Scientific activity	No publications: 41.8%; <5 publications: 41.1%; 5–30 publications: 12.8%; >30 publications: 4.3%
University teaching	Yes: 31.2%; No: 68.8%

## Data Availability

The original contributions presented in this study are included in the article. Further inquiries can be directed to the corresponding author.
